# Supplementary material for: Maternal prenatal screening programs that predict trisomy 21, trisomy 18, and neural tube defects in offspring
Source: PLoS One. 2023 Feb 21;18(2):e0281201. doi: 10.1371/journal.pone.0281201 (PMC9942960; doi:10.1371/journal.pone.0281201)
Supplement: S1 File — (PDF) [file pone.0281201.s003.pdf]

杭州市妇产科医院（杭州市妇幼保健院）
杭州市第一人民医院钱江新城院区

伦理项目审批表

【2021】医伦审 A 第（3）号-02

事件/项目名称：AFP-L2 联合早孕期非整倍体筛查标志物预测胎儿 Trisomy 21,18 和 ONTD 的风险模型建立及应用研究

报审科室：产前筛查实验室	申请人：陈益明	申请事项：科研项目
审查方式： 会议审查	审查时间：2021.6.2	会议地点：行政楼五楼会议室

伦理委员会联系人： 黄坚	联系电话： 0571-56005074

审查材料：研究方案、科研项目保密承诺书、主要研究者履历、其他资料

投票结果：伦理委员会 14 位成员对上述文件进行了认真的审查和讨论，并进行了投票表决，其中投票人数： 14 人，结果如下：

同意	作必要修正后同意	作必要修正后重审	终止或暂停已批准试验	不同意
(14)票	(0)票	(0)票	(0)票	(0)票

审查意见：

经本伦理委员会审查，该研究项目符合伦理要求，同意其开展下一步研究。

杭州市妇产科医院（杭州市妇幼保健院）

伦理委员会（盖章）

杭州市妇产科医院伦理委员会

主任委员签名：[illegible]

日期 ：2021 年 6 月 2 日
