# Supplementary material for: Maternal prenatal screening programs that predict trisomy 21, trisomy 18, and neural tube defects in offspring
Source: PLoS One. 2023 Feb 21;18(2):e0281201. doi: 10.1371/journal.pone.0281201 (PMC9942960; doi:10.1371/journal.pone.0281201)
Supplement: S2 File — (DOC) [file pone.0281201.s004.doc]

**Ethical project approval form**

【2021】 Medical Ethics Review A (3) - 02

| Event/project name：Risk models and application research of AFP-L2 combined aneuploidy screening markers in early pregnancy to predict fetal with Trisomy 21, 18 and ONTD | | | | | | |
| --- | --- | --- | --- | --- | --- | --- |
| Reporting department: Prenatal diagnosis and screening center | | Applicant: Yiming Chen | | | Application matters: Research project ethics review | |
| Mode of review: Meeting review | | Meeting time: 2021.6.2 | | | Meeting place: Administrative Building 5th Floor Meeting Room | |
| Ethics Committee Contact: Jian Huang | | | Contact number: 0571-56005074 | | | |
| Review materials: research proposal, scientific research project confidentiality commitment letter, main researcher's resume, other materials | | | | | | |
| **Voting results:** 14 members of the ethics committee carefully reviewed and discussed the above documents and voted on them. The number of voters was 14, and the results are as follows: | | | | | | |
| agree  (14) votes | agree with the necessary amendments  (0) votes | review after make necessary corrections  (0) votes | | terminate or suspend an approved trial  (0) votes | | disagree  (0) votes |
| Review opinion：  After review by the ethics committee, the research project met the ethical requirements and agreed to carry out the next research application.  Hangzhou Women’s Hospital  (Hangzhou Maternity and Child Health Care Hospital)  Ethics Committee (seal)    Signature of the chairman:  Date : 2021/06/02 | | | | | | |
