# Supplementary material for: Maternal prenatal screening programs that predict trisomy 21, trisomy 18, and neural tube defects in offspring
Source: PLoS One. 2023 Feb 21;18(2):e0281201. doi: 10.1371/journal.pone.0281201 (PMC9942960; doi:10.1371/journal.pone.0281201)
Supplement: Attachment — Submitted filename: Review comments.pdf [file pone.0281201.s005.pdf]

Thank you for your submission. It is good to have more data on the performance of prenatal screening programs for fetal diseases in China.

There is an extensive body of evidence in the medical literature that reports and analyzes the performance of the different screening modalities for aneuploidy in the first and second trimesters of pregnancy.

STUDY DESIGN:

The research subject is relevant, and the sample size is very good.

However, some issues stand out regarding study design:
- The authors decided to analyze the screening performance for both aneuploidy and neural tube defects together. Most of the published studies have analyzed these results separately.
- Another curious design decision was to include twin pregnancies together with singleton pregnancies when it is well known that the screening performance differs significantly in both groups. Moreover, even within twins, some factors can influence significantly the detection rate of aneuploidy and structural anomalies, such as chorionicity.
- for some reason, the performance of screening for trisomy 13 was not included in the analysis
- patients who only had serum biochemistry measurements in the first trimester were included in the analysis together with those who had combined screening. "Double screening" or biochemical screening in the first trimesters is currently considered suboptimal.

READABILITY:
Unfortunately, the manuscript lacks clarity, and the choice of words is not appropriate in many cases, which makes it difficult to follow. There are serious grammatical errors, including unfinished sentences (page 4, line 89) and typos (page 17, line 301, the author's name is “Palomaki”; line). I strongly recommend that the authors should consider rewriting it with the advice of a native English speaker.

METHODS:
There are also serious conceptual and methodological errors, such as saying that the nuchal translucency was evaluated following the FMF recommendations but then stating that NT thickness was measured

"between 15 and 20 weeks" and that "the fetus was normal when NT was 2.5mm".

The strategy used to screen for neural tube defects is unclear, since, for example, data on ultrasound findings in the first and second trimesters are not included (was intracranial translucency measured in all cases? In what percentage of the cases with FTS was an NTD detected or suspected sonographically? Were second-trimester sonographic markers (lemon and banana signs) evaluated in all cases? What was the sonographic detection rate?)

RESULTS:

Some of the reported results are probably erroneous and require revision because they are not in line with what is known from the literature.

Examples:
page 8, line 166: should say "the median maternal age"

page 8, line 167: "the proportion of gravidas with advanced maternal age was 0.55% ". In most populations, advanced maternal age (defined as maternal age of 35 o more at delivery) ranges between 30-60%, so this sounds strange.

Errors like these undermine the credibility of the results of the analysis.

The tables are not clear and irrelevant data such as the value of the chi-square statistic are reported. For a better understanding, they should be simplified.

DISCUSSION:

The authors reach conflicting conclusions with all the previous experience in aneuploidy screening, such as stating that the biochemical screening of the second trimester had a higher detection rate than the screening of the first trimester. This, together with the low detection rates achieved in the FTS, suggest that the latter was performed with an insufficient level of quality since in many validation studies, the detection rate of the combined screening ranges between 75-90%.

The authors state that “The result shoed that STS for trisomy 21 had the highest detection rats, followed by FSTCS and FTS” but then say that “there were no statistical differences in the detection rates of trisomy 21 among the 3 screening programs”, and later say that “we concluded that FSTCS was superior to FTS and STS screening”. These statements appear to be contradictory.

Rather than provide plausible explanations, the authors suggest that this could be due to " the tendency for gravidas of advanced maternal age in this region to participate more in STS and less in FTS” or issues related to sample size, demographic factors, or “screening indicators across laboratories”

CONCLUSION:
Based on the above, I consider that the manuscript cannot be accepted for publication in its current version and could only be considered in case of a major revision.
