# Supplementary material for: Maternal prenatal screening programs that predict trisomy 21, trisomy 18, and neural tube defects in offspring
Source: PLoS One. 2023 Feb 21;18(2):e0281201. doi: 10.1371/journal.pone.0281201 (PMC9942960; doi:10.1371/journal.pone.0281201)
Supplement: Attachment — Submitted filename: PLOS ONE-Response (PONE-D-22-00752).docx [file pone.0281201.s006.docx]

Dr. Editor

**PLOS ONE**

October 24, 2022

Dear editor

We would like to submit the enclosed manuscript PONE-D-22-00752 entitled “**Maternal prenatal screening programs that predict trisomy 21, trisomy 18, and neural tube defects in offspring**”, which we wish to be considered for publication in **PLOS ONE***.* We have made changes in response to journal requirements and reviewers' comments, and marked in red font, specific modifications are as follows. Our manuscript has also been linguistically twice retouched by International Science Editing and is presented in an intelligible fashion and in standard English.

**Journal Requirements:**

**Q1.** Please ensure that your manuscript meets PLOS ONE's style requirements, including those for file naming.

Answer1: We have revised the manuscript to meet PLOS ONE's style requirements.

**Q2.** Please include your amended Funding Statement within your cover letter. We will change the online submission form on your behalf.

**Answer2:** Because the original fund has not been formally issued, we modify the fund as follows: “This study was supported by Hangzhou Medicine and Health Science and Technology Research Project (2017A055) and Yiming Chen was chief investigator. There was no additional external funding received for this study”.

**Q3.** Your ethics statement should only appear in the Methods section of your manuscript. If your ethics statement is written in any section besides the Methods, please delete it from any other section.

**Answer3:** We have removed the ethics statement except for the Methods section.

**Q4.** Please include captions for your Supporting Information files at the end of your manuscript, and update any in-text citations to match accordingly.

**Answer4:** We have updated the Supporting Information files and in-text citations.

**Review Comments to the Author:**

Reviewer #1:

STUDY DESIGN:  The research subject is relevant, and the sample size is very good.  However, some issues stand out regarding study design:

**Q1:** The authors decided to analyze the screening performance for both aneuploidy and neural tube defects together. Most of the published studies have analyzed these results separately.

**Answer1:** Although most published studies have analyzed the screening performance of aneuploidy and neural tube defects separately for these outcomes. However, there is little relevant literature from Hangzhou, China.

**Q2:** Another curious design decision was to include twin pregnancies together with singleton pregnancies when it is well known that the screening performance differs significantly in both groups. Moreover, even within twins, some factors can influence significantly the detection rate of aneuploidy and structural anomalies, such as chorionicity.

**Answer2:** For twin pregnancies we usually recommend NT or other prenatal screening or prenatal diagnosis, but there is always a small percentage of twin pregnancies that voluntarily opt for serological screening, so we also include twin pregnancies together with singleton pregnancies in our analysis.

**Q3:** for some reason, the performance of screening for trisomy 13 was not included in the analysis

**Answer3:** Since only 3 cases of trisomy 13 were detected in this cohort, we did not list trisomy 13 separately, but included it among the other abnormalities.

**Q4:** patients who only had serum biochemistry measurements in the first trimester were included in the analysis together with those who had combined screening. "Double screening" or biochemical screening in the first trimesters is currently considered suboptimal.

**Answer4:** Although "Double screening" or biochemical screening in the first trimesters is currently considered suboptimal, some successes have been achieved since the mid-pregnancy duplex screening program was included in the local financial support for the prevention and control of major birth defects in Hangzhou, China.

READABILITY:

**Q5:** Unfortunately, the manuscript lacks clarity, and the choice of words is not appropriate in many cases, which makes it difficult to follow. There are serious grammatical errors, including unfinished sentences (page 4, line 89) and typos (page 17, line 301, the author's name is “Palomaki”; line). I strongly recommend that the authors should consider rewriting it with the advice of a native English speaker.

**Answer5:** Thanks to the reviewers and responsible editors for your corrections. The language and grammar of this manuscript have been revised by the relevant English professionals.

METHODS:

**Q6:** There are also serious conceptual and methodological errors, such as saying that the nuchal translucency was evaluated following the FMF recommendations but then stating that NT thickness was measured "between 15 and 20 weeks” and that “the fetus was normal when NT was 2.5mm”.

**Answer6:** Thanks to the reviewer's correction. We wrote wrongly at that time, and it should be 11-13+6 weeks for NT thickness check, "the fetus is normal when NT is <2.5 mm".

**Q7:** The strategy used to screen for neural tube defects is unclear, since, for example, data on ultrasound findings in the first and second trimesters are not included (was intracranial translucency measured in all cases? In what percentage of the cases with FTS was an NTD detected or suspected sonographically? Were second-trimester sonographic markers (lemon and banana signs) evaluated in all cases? What was the sonographic detection rate?)

**Answer7:** Among 108118 pregnant women, only a small proportion of those with a high risk of NTD by prenatal screening or low risk of NTD were evaluated for mid-pregnancy ultrasound markers (lemon and banana signs).

There were 3044 cases of confirmed abnormal findings after prenatal diagnosis and ultrasound, of which 56 cases were NTD. The ultrasound detection rate was 2.82%.

RESULTS:

**Q8:** Some of the reported results are probably erroneous and require revision because they are not in line with what is known from the literature. Examples: page 8, line 166: should say “the median maternal age”

**Answer8:** Thanks to the reviewers and responsible editors for their corrections. We have revised page 8, line 166 to "the median maternal age".

**Q9:** page 8, line 167: “the proportion of gravidas with advanced maternal age was 0.55% “. In most populations, advanced maternal age (defined as maternal age of 35 o more at delivery) ranges between 30-60%, so this sounds strange.

**Answer9:** Since advanced maternal age is not recommended for FTS in early pregnancy in our region, so the advanced maternal age was 0.55%, while ISTCS advanced maternal age was 22.80%, so the participation in FTS and FSTCS is basically for low age pregnant women. The advanced maternal age in ISTS was 22.80%. The above are the difference between our study and others.

**Q10:** The tables are not clear and irrelevant data such as the value of the chi-square statistic are reported. For a better understanding, they should be simplified.

**Answer10:** We modified and simplified the tables accordingly, e.g. deleting the columns of chi-square statistics in Table 1 and the columns of Z statistics in Tables 5-6.

DISCUSSION:

**Q11:** The authors reach conflicting conclusions with all the previous experience in aneuploidy screening, such as stating that the biochemical screening of the second trimester had a higher detection rate than the screening of the first trimester. This, together with the low detection rates achieved in the FTS, suggest that the latter was performed with an insufficient level of quality since in many validation studies, the detection rate of the combined screening ranges between 75-90%.

**Answer11:** In our large retrospective cohort study, the detection rate of biochemical screening for midterm pregnancy alone was higher than that of early pregnancy screening, whereas the detection rate of biochemical screening for midterm pregnancy alone was lower than that of early pregnancy screening in low-risk pregnant women. As for the low detection rate of FTS, it may be related to statistical bias because nearly 1/3 of the pregnant women in the study missed early pregnancy screening.

**Q12:** The authors state that “The result shoed that STS for trisomy 21 had the highest detection rats, followed by FSTCS and FTS” but then say that “there were no statistical differences in the detection rates of trisomy 21 among the 3 screening programs”, and later say that “we concluded that FSTCS was superior to FTS and STS screening”. These statements appear to be contradictory.

**Answer12:** The results of this study showed that ISTS had the highest detection rate for trisomy 21 in 22.80% of high-risk pregnancies, followed by FSTCS and FTS". In conclusion, FSTCS was superior to FTS and ISTS screening and greatly reduced the number of high-risk pregnancies for trisomy 21 and 18. FSTCS reduced the high-risk positive rate for chromosomal abnormal disorders. It is possible that the reviewers did not understand that we mainly emphasize that there is a difference between the different screening protocols for screening high-risk positive rates for the previous, but no difference for the final confirmation of trisomy 21 and 18. STS here refers to ISTS. See Figure 1.

**Q13:** Rather than provide plausible explanations, the authors suggest that this could be due to " the tendency for gravidas of advanced maternal age in this region to participate more in STS and less in FTS” or issues related to sample size, demographic factors, or “screening indicators across laboratories”

**Answer13:** Due to some policies related to prenatal screening in China, older pregnant women are usually advised to undergo direct mid-trimester screening. Therefore, older pregnant women in the region tend to be more involved in ISTS. STS here refers to ISTS. See Figure 1

We confirmed that this manuscript has not been published elsewhere and is not under consideration by another journal. All authors have approved the manuscript and agree with submission to **PLOS ONE.** The authors have no conflicts of interest to declare.

We shall look forward to hearing from you at your earliest convenience.

* Corresponding author:

Yiming Chen

Department of Prenatal Diagnosis and Screening Center Hangzhou Women’s Hospital (Hangzhou Maternity and Child Health Care Hospital), No. 369, Kunpeng Road, Shangcheng District Hangzhou, Zhejiang 310008, China

[TEL: +86](TEL:+86) 56005843

E-mail: cxy40344@163.com

Yours sincerely.

Yiming Chen
