# Supplementary material for: Maternal prenatal screening programs that predict trisomy 21, trisomy 18, and neural tube defects in offspring
Source: PLoS One. 2023 Feb 21;18(2):e0281201. doi: 10.1371/journal.pone.0281201 (PMC9942960; doi:10.1371/journal.pone.0281201)
Supplement: Attachment — Submitted filename: Response letter.docx [file pone.0281201.s007.docx]

Response letter

Dear editor:

Our paper “Maternal prenatal screening programs that predict trisomy 21, trisomy 18, and neural tube defects in offspring” (PONE-D-22-00752R1), has been modified according to the reviewer's suggestion, and the modified part is marked with yellow shade.

Reviewer #1: Thank you for sending a revised version of the paper. The text has improved significantly in clarity. Tables are also easier to understand in this new version.

Please consider the following edits:

Page 4 line 91: "history" of diabetes

Page 6 line 134: "echolucent" instead of "transparent"

Page 17 line 243: "ductus venosus" instead of "venous catheter"

Response: Thanks for your advice, we have made modifications to these parts with shaded in yellow.

Reviewer #2: I read with great interest the Manuscript titled “Maternal prenatal screening programs that predict trisomy 21, trisomy 18, and neural tube defects in offspring” (PONE-D-22-00752R1), which falls within the aim of this Journal.

In my honest opinion, methodology is accurate and conclusions are supported by the data analysis.

Nevertheless, authors should clarify some point and improve the discussion citing relevant and novel key articles about the topic.

Authors should consider the following recommendations:

- I suggest to improve the intelligibility and fluidity of the discursive part: in particular the presentation of the results and the conclusions are sometimes ambiguous or not clear (e.g. where there are sentences such as "the differences were statistically significant" should be specificated between which groups).

Response: Thanks for your advice, we have amended the presentation of the results and the conclusions are sometimes ambiguous or not clear.

- I recommend the authors to specify the meaning of the abbreviations before using them (line 59 : NTD),

to correct the word "medium" with "median" (line 141) and to add "age" after "gestational" (line 186)

Response: Thanks for your advice, we have made modifications to these parts with shaded in yellow.

- I suggest to the Authors to specificate the age range considered "AMA" in table 4 and to add "fetuses" after "healty" at line 338.

Response: Thanks for your advice, AMA age range was 35 years or older, we have revised this, and we have added "fetuses" after "healthy" in text.

- In the Results section, the Authors have simply reported the p values, from which however it is not possible to deduce the real clinical relevance of the highlighted statistical significance. In order to better understand the obtained results, I suggest reporting not only the p values, but also the corresponding confidence intervals

 Response: Thanks for your advice, we put confidence intervals in the tables concerned.

- In the discussion section I could not find considerations about neural tube defects.

Response: Thanks for your advice, we have added considerations about neural tube defects. NTD is a serious neurological defect of the fetus, causing a great challenge to the families and societies [25]. Currently, the diagnosis of ONTD mainly relies on ultrasound images and serum AFP in the second trimester in the maternal serum [25]. Our study showed that the detection rate of NTD in FTS were higher than in ISTS, which may be because that NT examination improved the detection rate of FTS. Therefore, NTD screening should be combined with ultrasound images and maternal serum screening. In addition, Chen et al. [26] Showed that AFP variants (AFP-L2 and AFP-L3) are favorable biomarkers for screening ONTD, and it have superior sensitivity and specificity. [Gerardo](https://pubmed.ncbi.nlm.nih.gov/?term=Sep%C3%BAlveda-Gonz%C3%A1lez+G&cauthor_id=33266467) et al. [27] showed that abnormal intracranial translucency during the first-trimester may be a useful screening marker for early detection of NTDs.

- It is important to report the results obtained by the authors in the context of clinical practice and to adequately highlight the strengths and the contribution this study adds to the literature already existing on the topic and to future study perspectives.

Response: Thanks for your advice, we have added some other viewpoint with shaded in yellow.

Undeniably, NIPT, as a non-invasive, highly sensitive and specific feature, has become a novel screening method for fetal chromosome malformations. With the rapid development of molecular biology, perhaps one day in the future, the traditional maternal serum biochemical screening method may be a secondary screening method.

Although compared to ISTS, FSTCS did not significantly improve the rate of chromosome disease detection. However, for eligible pregnant women, we still recommend FSTCS, because it will reduce the high risk of chromosomal diseases and reduce the probability of invasive diagnosis in pregnant women.

- Does this manuscript conform the Enhancing the QUAlity and Transparency Of health Research (EQUATOR) network guidelines? It would be mandatory to declare about this element

Response: This article was conformed the Enhancing the QUAlity and Transparency Of health Research (EQUATOR) network guidelines. We have added it in the text.

- I could not find any information regarding the approval of the Institutional Review Board. Did author this approval before the study start?

Response: Thanks for your advice, It is located in the Subjects and Methods.

- I recommend to highlight, at least briefly, the role of absolute risk for the detection of fetal aneuploidies in the first-trimester screening and the impact of advanced maternal age (authors may refer to: PMID: 27442264; PMID: 25027820).

Response: Thanks for your advice, we have added some other statement with shaded in yellow.

Studies have shown that advanced maternal age is associated with adverse pregnancy outcomes, such as preterm birth, miscarriage, and fetal chromosome abnormalities [28]. In this study, we found that the incidence of trisomy 21 and trisomy 18 was higher in advanced maternal age than in non-advanced maternal age. The detection rate for fetal chromosomal malformations of advanced maternal age in ISTS was higher than that of non-advanced maternal age. But studies have showed that the effect of advanced maternal age on fetal chromosome abnormalities has been overestimated, introducing absolute risk (eliminating the mother's age risk) from the risk algorithm may reduce the screening positive rate for advanced maternal age and increase the screening positive rate for non-advanced maternal age [29].
